# Motivation for Behavior Change among Women with Recent Gestational Diabetes and Their Partners—A Qualitative Investigation among Participants in the Face-It Intervention

**DOI:** 10.3390/nu15183906

**Published:** 2023-09-07

**Authors:** Anne Timm, Karoline Kragelund Nielsen, Helle Mölsted Alvesson, Dorte Møller Jensen, Helle Terkildsen Maindal

**Affiliations:** 1 Health Promotion Research, Copenhagen University Hospital—Steno Diabetes Center Copenhagen, 2730 Herlev, Denmark; karoline.kragelund.nielsen@regionh.dk (K.K.N.); htm@ph.au.dk (H.T.M.); 2 Department of Public Health, Aarhus University, 8000 Aarhus, Denmark; 3 Department of Global Public Health, Karolinska Institutet, 17177 Stockholm, Sweden; helle.molsted-alvesson@ki.se; 4 Steno Diabetes Center Odense, Odense University Hospital, 5000 Odense, Denmark; dorte.moeller.jensen@rsyd.dk; 5 Department of Gynaecology and Obstetrics, Odense University Hospital, 5000 Odense, Denmark; 6 Department of Clinical Research, Faculty of Health Sciences, University of Southern Denmark, 5000 Odense, Denmark

**Keywords:** gestational diabetes, health promotion, behavior change, process evaluation, diabetes prevention, diet, physical activity, intervention, couple interviews

## Abstract

Promoting diet and physical activity is important for women with recent gestational diabetes mellitus (GDM) and their partners to reduce the risk of future type 2 diabetes (T2D). The study aimed to understand how motivation for changing diet and physical activity behaviors among women with recent GDM and their partners was experienced after participation in the Danish Face-it intervention. Fourteen couples’ interviews were conducted. Data analysis followed a reflexive thematic analysis. Guided by self-determination theory and interdependence theory, we identified four themes affecting couples’ motivation for health behavior change: (1) The need to feel understood after delivery; (2) adjusting health expectations; (3) individual and mutual preferences for health behaviors; and (4) the health threat of future T2D as a cue to action. We found that couples in general perceived the Face-it intervention as useful and motivating. Using couple interviews increased our understanding of how the women and partners influenced each other’s perspectives after a GDM-affected pregnancy and thus how targeting couples as opposed to women alone may motivate health behavior change.

## 1. Introduction

Gestational diabetes mellitus (GDM) affects 14% of all pregnancies globally [1] and is the most common complication in pregnancy [2]. In Denmark, 6% of all pregnancies are affected by GDM [3]. Although usually a transient condition, GDM is associated with an increased risk of short- and long-term adverse health outcomes in both women and their offspring [1,4]. Also, the risk of recurrent GDM is high, ranging between 30 and 84% [5]. In the long term, women with prior GDM and their offspring are at an increased risk of developing type 2 diabetes (T2D) [6,7,8], and body mass index (BMI) remains the main modifiable risk factor for T2D development among women with prior GDM [9]. Structured interventions targeting weight loss have shown that reducing T2D risk is possible through changes in diet and physical activity in this group of women [10,11]. Also, persistent changes in diet and physical activity have been found to uphold T2D risk reduction after 10 years among women with prior GDM [12]. Still, it remains unclear how health behaviors can be sustained in the long term [13]. According to a meta-analysis of interventions applying self-determination theory, some types of motivation increase the likelihood of sustained behavior change across target populations and behaviors [14]. Thus, motivation is most likely an active mechanism in seeking to explain the maintenance of healthy behavior [15]. Identifying such mechanisms of change is essential to understanding how intervention effects have occurred and how an intervention may be replicated in other settings [16]. Thus, investigating motivation as an assumed mechanism for health behavior change is important to understand how prevention efforts targeting T2D risk may be improved.

Many qualitative studies have shown that social support from a partner is a key motivational factor for improving health behaviors among women with prior GDM [17], even in the Danish context [18]. Still, few interventions have focused on the family setting, which may explain the limited success of interventions in demonstrating long-term behavior change. Few interventions have involved women with prior GDM and their partners [19,20]. McManus et al. showed that when these women and their partners participated together, women’s retention in the intervention increased, and when their partners lost weight, the women’s weight tended to follow [19]. Furthermore, Brazeau and colleagues documented that physical activity levels increased in both women with prior GDM and their partners after engaging in an intervention including mutual physical activity and meal sessions [20]. For both interventions, retention among couples remained high [19,20]. Couples often share daily routines in the household and health behaviors, which most likely results in them influencing each other’s health status [21,22]. This may explain why partners of women with prior GDM have also been found to be at increased risk of T2D [8,23]. This conjoint risk [24] suggests the need for directing health interventions at both parties [25]. Digital technology, e.g., text messages, seems to increase retention, acceptability, and health behavior outcomes of interventions among postpartum women [26,27] and women with prior GDM [28]. Still, a knowledge gap exists on how health promotion interventions influence motivation for health behaviors among women with prior GDM and their partners.

### 1.1. The Face-It Intervention

The Face-it health promotion intervention was developed to reduce the risk of T2D and increase the quality of life in women with prior GDM and their families. The effectiveness of the intervention is being evaluated in a randomized controlled trial, and details are published elsewhere [29,30]. Women with GDM and their partners were recruited from 2019 to 2022 through obstetric departments in the three largest cities in Denmark, where the intervention was delivered. Women could participate in the trial regardless of whether they had a partner or not. Thus, women without a partner and women with partners who declined to participate could participate alone. If both the woman and her partner accepted participation and the couple was randomized to the intervention group, the partner was invited to all intervention activities.

**Figure 1:**
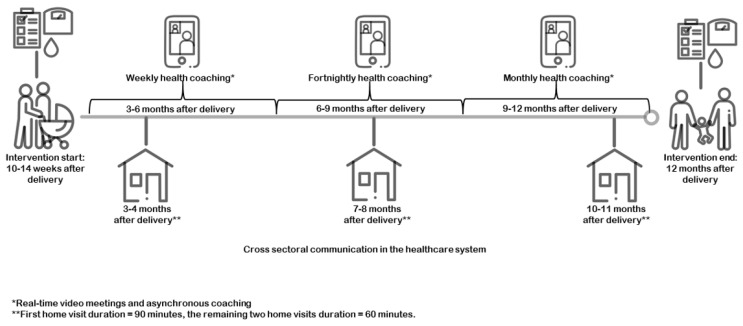
Overview of the planned delivery of the Face-it intervention [32].

The Face-it intervention commenced 10–14 weeks after delivery and continued until the baby was around 12 months old. The intervention was based on three major components. The first component comprised active involvement of health visitors in addition to usual care through three home visits supported by a dialogue tool “the family wheel”, which addressed five topics: GDM and risk/prevention of T2D; daily routines; food and meals; exercise/movement; and family, friends, and network (Figure S1). Municipality-based health visitors (nurses trained in newborn health and family wellbeing) delivered the home visits. The second component consisted of digital health technology. This technology included (a) goal setting, (b) real-time meetings, and (c) asynchronous coaching (text and video). Trained health coaches (primarily health visitors) delivered digital health technology through the digital platform “the LIVA app” [31]. The LIVA app included customizable materials for health coaches to personalize guidance for families, encouraging healthy behaviors. The third intervention component involved communication and coordination across health sectors to ensure that couples were provided with coherent information in the postpartum period. The three home visits were planned to be delivered at fixed time points, coordinated in collaboration between the couples and the health visitor. Digital health coaching was delivered with varying intensity across the nine months according to the needs and preferences of the family (see Figure 1).

The healthcare professionals delivering the intervention received education about GDM and training on communicating about GDM, including the care pathway and the risk of T2D. Also, they were supported in embedding a health-promoting perspective as defined by the World Health Organization (WHO) Europe, i.e., empowering, participatory, holistic, equitable, and multi-strategy [33]. For example, healthcare professionals were trained to focus on social support and well-being as pre-requisites for health behavior change and to be sensitive to the family’s situational needs. This education was intended to enable healthcare professionals to promote health in general as well as physical activity, healthy dietary behaviors, and breastfeeding through the following mechanisms: social support, motivation, self-efficacy, risk perception, and health literacy. To improve our understanding of how the Face-it intervention may induce changes in health behaviors among couples after participation, we explored motivation as an assumed mechanism.

### 1.2. Self-Determination Theory and Interdependence Theory

Theory-driven evaluation designs are recommended when investigating mechanisms of change in interventions to better understand intervention effects [34]. To advance our understanding of how couples’ motivation for health behavior changes was affected by the Face-it intervention, we applied the self-determination theory (SDT) [35] and the interdependence theory [36].

SDT is a theoretical perspective on human motivation that highlights humans’ inner resources for personality development and behavioral self-regulation [35]. In this study, SDT was used in the analysis to understand women’s and their partners’ motivation for health behavior change. SDT distinguishes between extrinsic and intrinsic motivation. Intrinsic motivation is characterized by actions driven by a personal interest in practicing a behavior, e.g., joy, whereas extrinsic motivation is driven by external factors such as rewards, obligation, social acceptance, and individual goals and values. SDT posits that the more internalized a motivation is to perform a behavior, the more likely the individual is to sustain it. According to SDT, *relatedness*, *perceived competence*, and *autonomy* are underlying personal attributes that contribute to the internalization of individual motivation. Relatedness concerns the need to feel understood through the establishment of a non-judgmental, positive, and empathic environment. Perceived competence refers to one’s experienced ability to perform a behavior. SDT posits that motivation becomes more autonomously driven when individuals experience relatedness and perceived competence. Thus, autonomy involves the need to consider oneself the driver of one’s own behaviors [35].

The interdependence theory developed by Lewis et al. is a dyadic theory that can be used to understand each partner’s perspective, thereby revealing both individual and collective influences on health behaviors [36]. Interdependence theory was applied in the analysis to investigate couples’ mutual motivation for health behavior change. The main hypothesis behind interdependence theory is that mutual support within the relationship is the most effective way to sustain health behaviors and that when health behaviors are perceived as meaningful to the couple and their relationship, the incentive for them to perform these behaviors increases. Lewis et al. incorporate pre-dispositional factors, which influence couples’ motivation for health behavior change. *Preferences for outcomes* refer to the extent to which the couples agree on project goals in terms of performing health behaviors within couples. Another pre-dispositional factor is couples’ interpretation of *health threats*, which, in the case of GDM, is how couples’ perceive the risk and consequences of T2D as a cue to preventive action [36]. Interdependence theory includes three other factors: *relationship functioning*, *communication style*, and *gender*, which were less evident in the data and thus absent in the results.

## 2. Materials and Methods

### 2.1. Study Design and Setting

The current study followed a qualitative interview-based design and contributes to the process evaluation of the Face-it intervention [29].

In Denmark, screening for GDM is based on selected risk factors, including pre-pregnancy BMI ≥ 27 kg/m^2^, family history of diabetes, previous birth of a child with a birth weight ≥ 4500 g, glucosuria, polycystic ovary syndrome, and multiple pregnancies [37]. According to current Danish guidelines, pregnant women are diagnosed with GDM by a 2 h, 75 g oral glucose tolerance test with a diagnostic glucose threshold of ≥9 mmol/L (venous plasma or capillary blood) or ≥10 mmol/L (capillary plasma) [38]. After diagnosis, women are advised about diet, physical activity, and self-monitoring of blood glucose, and if this treatment is not sufficient to prevent high blood glucose levels, insulin treatment is started [39]. Due to the risk of fetal and birth complications, the women are closely monitored by a multidisciplinary team including midwives, obstetricians, nurses, dieticians, and endocrinologists [40]. Following childbirth, Danish guidelines recommend consistent screening for T2D at 4–12 weeks postpartum, with subsequent screenings scheduled every 1–3 years with their general practitioner [39]. Additionally, they advise providing counseling on dietary habits and physical activity to mitigate the women’s risk of developing T2D in the future. However, diabetes screening uptake is low among women with prior GDM, with only 17% attending screening after 4–6 years [41]. Nonetheless, no other systematic follow-up or preventative initiatives exist in Denmark.

### 2.2. Data Collection

The first author, AT, conducted semi-structured couple interviews with women with recent GDM and their partners. All women and their partners who had completed the Face-it intervention within the last year, between January 2020 and January 2021, were invited to participate through a written information letter sent via a secure digital platform (e-Boks). Non-respondent couples received a reminder text message approximately four weeks after the invitation. AT telephoned the couples who agreed to participate to schedule an interview. The majority of interviews were carried out in the homes of the participants [42]. However, due to restrictions during the COVID-19 pandemic, four interviews were performed online. All interviews were conducted between mid-November 2021 and mid-February 2022 and were performed in Danish.

Of the 27 couples invited, 14 agreed to participate in an interview. Nine couples did not respond, and four couples declined participation. Two couples indicated disinterest, and two couples lacked time and energy to participate. The characteristics of the couples participating in interviews are presented in Table 1.

AT, a female researcher in her late twenties with a background in public health science, conducted all interviews. Prior to this study, AT was involved in the development of the Face-it intervention and, among other things, participated in the education of healthcare professionals delivering the intervention. Moreover, AT had also conducted interviews with the intervention deliverers in the early stages of implementation [43]. Thus, AT was highly integrated in the intervention, which she actively leveraged in this study to develop and challenge her assumptions about the impact of the intervention on couples’ motivation for health behavior change. AT had no contact with the couples prior to inviting them to participate in the interviews for this present study.

An interview guide was developed by AT and discussed with KKN and HTM, as well as with other researchers in the Face-it Study. An explorative approach focused on motivation was employed. The interview guide included questions about parenthood, perceptions of health, experiences with home visits, and digital health coaching (see Table S1). All interviews were audio recorded and/or online video recorded. The interviews lasted between 48 and 79 min.

Before the interviews were initiated, the couples were informed that the interviews would be used to provide knowledge to strengthen care for families in which the woman had GDM. During the interviews, an overview of the intervention activities (Figure 1) and the family wheel (Figure S1) were used as prompts to help women and partners recall their experiences with the intervention. The woman and partner were asked to write down their respective perceptions of the ‘useful’ and ‘less useful’ aspects of the intervention (Table S1). The woman or partner who said the least was asked to provide her/his reflections first to ensure their engagement in the interview. This exercise was not included in interviews performed online, and the presence of a child made it difficult for the couple to reflect separately on their answers.

### 2.3. Data Analysis

Data analysis followed an abductive approach using reflexive thematic analysis, in which the researcher’s position is continuously reflected upon to advance analysis [44,45]. Interviews were transcribed verbatim by AT or a research assistant. Initially, AT listened to all audio recordings to familiarize herself with the data and then coded all transcripts inductively. During the first reading of the empirical data, AT explored couples’ perspectives on health behavior change during and after participating in the Face-it intervention. During this process, AT coded both assumed and unintended mechanisms of change, e.g., “changes in resources after having a baby” or “perception of T2D risk”. In the second coding of the empirical data, AT identified patterns related directly to motivation and thereafter looked for theories to complement and nuance the data. The second round of coding was guided by SDT and interdependence theory [35,46]. Themes and subthemes were created during this phase. Example quotes were translated to English and confirmed by a native English speaker and the co-authors. Nvivo v12 was used to structure the coding process [47]. Examples of the data analysis process are presented in Supplementary Table S2.

### 2.4. Ethical Considerations

The Face-it trial is registered with ClinicalTrials (gov NCT03997773). The woman and her partner gave written consent to participate through the Danish digital platform (e-Boks) after receiving information on study aim, pseudo-anonymity, and voluntary participation. Consent for audio and/or video recordings was obtained prior to the interviews. During couple interviews, staying sensitive to couple dynamics is important, and therefore, AT abstained from going into discussions that might negatively affect the couple’s relationship [48]. The study is reported according to the Consolidated Criteria for Reporting Qualitative Research (COREQ) [49].

## 3. Results

We identified the four following themes related to couples’ motivation for health behavior change: (1) the need to feel understood after delivery; (2) adjusting health expectations; (3) individual and mutual preferences for health behaviors; and (4) the health threat of future T2D as a cue to action.

### 3.1. The Need to Feel Understood after Delivery

In the first theme, we find that couples’ experiences of feeling understood by the healthcare professionals delivering the Face-it intervention affected their motivation for health behavior change. The couples described their everyday lives as characterized by a limited ability to be spontaneous, time restrictions, and an increased need for collaboration to ensure the baby’s wellbeing. Due to these requirements, many participants expressed that they would not have accepted participation if it had required them to leave their homes. Also, most participants described it as critical that the hardship they faced caring for a baby be acknowledged in their interaction with the healthcare professionals.

*“We had some good talks about planning and what we wanted to do using the family wheel [dialogue tool used in the home visits], especially that sometimes you need to accept that the energy just isn’t there. And how you’re not supposed to rearrange your life at this time but rather use this period to regain energy.”*

(Woman, Couple 11)

For instance, several couples stated that they experienced a need to relax to regain energy, e.g., by watching television after the baby was put to bed. When healthcare professionals recognized such needs as well as the emotional burden of being a parent, including the feeling of guilt of not living up to societal expectations of “proper” parenthood, most couples reported feeling understood by the healthcare professional. In particular, advice given by healthcare professionals on family habits, e.g., meal planning, household chores, and delegation of assignments between couple members, was mentioned by couples as enabling them to rethink their habits in a positive and non-judgmental way. Also, many couples stressed the importance of discussing coping strategies with the healthcare professional to relieve their stress, sleep deprivation, and emotional burden:

*“I’m glad I took part in [the intervention] because obviously one has learned something. It’s not all about exercising and what one eats. It’s about feeling good at the same time. When [name of healthcare professional] suggested that I should insert a goal into the LIVA app reminding me to relax—that was just amazing instead of having a bad conscience about everything I didn’t do.”*

(Woman, Couple 10)

However, couples noted that the LIVA app sometimes compromised the supportive environment established with the healthcare professional when they received feedback on the app that could be perceived as impersonal and auto-generated. As a consequence, couples mentioned instances of performative reporting in the LIVA app that did not match their actual goals or health behaviors. Also, the LIVA app was unable to automatically synchronize with their Android-based smartphones. This meant that couples had to manually register their health behaviors and goals into the app, which was perceived to be incompatible with the busy everyday life of the family. Motivated by external factors, e.g., registering in the app to please the health coach, couples’ motivation to comply with the advice decreased.

*“If I hadn’t registered for a week, then I had to sit and scroll back for each day, and I simply didn’t want to continue. I thought it was too much trouble.”*

(Partner, Couple 5)

### 3.2. Adjusting Health Expectations

The second theme deals with how feelings of competence and autonomy among the couples affected their motivation to engage in healthy dietary and physical activity behaviors. Most couples equated a healthy meal with a meal that was homecooked, and physical activities concerned “putting on a sports bra or running shoes”. Lack of time was often described by couples as the main barrier to conducting such health behaviors. However, many couples described how the healthcare professionals in the Face-it intervention broadened their views on the kind of behavior changes that could be considered health-promoting. This new understanding of how even small changes might add up and positively affect health was accompanied by an expressed increase in perceived competence, which in turn internalized their motivation to perform health behaviors.

*She [healthcare professional] said “count everything when you talk about physical activity. It’s okay to go out in the garden and pick tomatoes or do garden work for 5 min. Walk to the grocery store. Take the stairs. If you spend 15 min pram walking, that’s fine”.*

(Partner, Couple 4)

Furthermore, some couples described being encouraged by the healthcare professional to keep performing their preferred physical activities. As a result, couples were more likely to practice that behavior because it resonated with their current interests, e.g., walking. Getting support from healthcare professionals to identify alternative solutions to unhealthy habits was also described as useful for couples. As an example of a behavioral change adopted after participating in the intervention, one couple mentioned buying cans of carbonated soft drinks instead of 1.5 L bottles, and another couple stated:

*Partner: We used to have a fast-food Friday with food from the grill or McDonalds. In a period where we had limited energy, we talked to her [healthcare professional] about finding fast-food alternatives.*

*Woman: Now, we can have a fast-food day with homemade pizza with salad and less cheese and more vegetables. So, healthier alternatives, but we still call it fast-food day.*

(Couple 12)

Couples also reported situations during which their motivation to perform health behaviors became more externalized. Some couples considered the advice on health behaviors inappropriate because their own health was not considered a priority while they had a small baby. Some couples described the advice from healthcare professionals as prescriptive in the sense that they felt that the healthcare professional told them what to do without considering their family’s current resources, needs, and preferences. Consequently, these couples expressed feelings of low autonomy and support from the healthcare professional, which externalized their motivation to improve diet and physical activity behaviors.

*“I think she [the healthcare professional] was a tough lady. She made some suggestions, but they weren’t always realistic. I don’t know if they have too many families [in the intervention] but she didn’t really consider who we are. I felt like she said, “just eat cabbage”, but with a small child and a husband who works a lot, it’s not that easy.”*

(Woman, couple 7)

External motivation was also underscored when some women mentioned that they initially participated in the intervention mainly to support research rather than to change health behaviors themselves.

### 3.3. Individual and Mutual Preferences for Health Behaviors

The third theme focuses on how concordant and discordant preferences among couples affect their individual and mutual motivation to perform health behaviors. Due to the demands of the child, couples viewed their family and its functionality as their main priority, which took precedence over individual health behaviors. Performing health-related activities with their children as a family was a shared priority among most couples. Experiencing support and advice from healthcare professionals on planning family-based activities, which focused on family well-being, increased their mutual motivation for engaging in such activities despite the competing demands of everyday chores, as portrayed by Couple 14:

*Woman: We talked to the healthcare professional about wanting to go out more—like a walk in the woods. Not necessarily for the sake of being physically activity, but just as much for the fresh air and energy and the kids’ enjoyment.*

*Partner: It reminded us that even though we’re busy and we should also set up an office and clean up the kitchen and stuff like that. No! We’ll have to go outdoors for everyone’s sake.*

As such, it seemed that couples’ mutual motivation increased when health behaviors were performed as a family, since this made them more fun. However, couples also expressed divergent individual preferences for engaging in health behaviors. Women were more likely than their partners to express their preference for spending time with their child over their interest in performing individual health behaviors. As one woman put it:

*“What if I had spent time at the gym, which I could have spent better with my children?”*

(Woman, Couple 10).

In addition to wanting to engage in family-based activities, partners also described an interest in individual activities or activities with their child(ren) without their spouse. Some couples, although mainly the partners, had successfully used digital health coaching through the LIVA app to increase their personal focus on weight loss, step counts, etc. and found the possibility of individual support appealing.

*“When the opportunity presents itself, I take the pram with great pleasure and walk down to pick him [baby] up from the nursery instead of taking the car. Then it’s just us time. I hadn’t thought about that before [the intervention] in the same way. It’s healthy for me and for us that these healthcare professionals have rattled us in a well-intentioned way.”*

(Partner, Couple 7)

### 3.4. The Health Threat of Future T2D as a Cue to Action

The fourth theme addressed how couples cognitively and emotionally respond to their risk of T2D diabetes and its perceived impact on their motivation for health behavior change. Most couples expressed being aware of the women’s future T2D risk. It was also evident that couples’ motivation was affected very differently by how they interpreted the health threat. Many women indicated that the GDM diagnosis was linked to uncertainty because it was unclear to them why they had developed GDM. The healthcare professionals often calmed the women and couples by trying to explain GDM as a diagnosis triggered by genes. These explanations seemed to calm the women and remove the guilt some women felt towards receiving the GDM diagnosis. The couples expressed how demystifying the GDM diagnosis increased their motivation to pursue health behaviors to reduce their T2D risk.

*“My first thought was, what could I have done differently? What did I do wrong? The fact that someone tells you that this [GDM] is just something the body does—and it’s [the risk] more pronounced among some women and some are placed in the GDM category.”*

(Woman, Couple 2)

The majority of couples acknowledged the fact that changing their health behaviors might reduce their future T2D risk, which in turn increased their motivation for health behavior change. The fact that the child was also at increased risk of developing T2D was of great concern to the couple. This information acted as a cue to action for health behavior engagement due to the importance couples attributed to being a healthy family. Nonetheless, some couples perceived it as unrealistic to promote their health behaviors while having a small baby.

*“When the baby is out, you relax a bit. It’s hard. You don’t get the sleep you need and you’re tired and all those things and you’re overwhelmed by emotions, then it’s not carrots in the fridge you think about.”*

(Woman, Couple 1)

Others described the development of GDM as a natural bodily response to pregnancy. In these situations, couples’ motivation for health behavior change seemed to be negatively affected by their belief that their risk of future T2D diabetes would be unaffected by changing their health behaviors.

*Woman: Diabetes runs in the family. So, it wasn’t because of anything else that I developed gestational diabetes.*

*Partner: Yes, it was actually really random.*

In general, the way in which the women interpreted their own risks affected their partners’ motivation to support them by engaging in health behaviors. For example, when knowledge of T2D risk motivated the women to increase their focus on health behaviors, their partners tended to become supportive, which contributed to a mutual motivation for health behavior change.

*You [woman] had figured out what it really was—what gestational diabetes meant and what you could do to prevent it—and you told me many times that it’s not just you it affects. It’s also [baby]. And then I thought “Okay, this is the way we need to go.”*

(Partner, Couple 3)

Though most partners acknowledged the woman’s and child’s risk of T2D, none of the partners mentioned being at risk of diabetes themselves.

*“Of course, it’s mostly the woman’s body, which is affected. It’s not about my body.”*

(Partner, Couple 2)

Rather, partners considered their participation in the intervention to be highly relevant with regard to supporting health behaviors and thereby reducing the risk of diabetes in women and children.

## 4. Discussion

Our study investigated motivation as an assumed mechanism for health behavior change among couples after participating in the Face-it intervention. We found that couples experienced a need to feel understood by the healthcare professional, which affected their motivation to engage in health behaviors. Further, when the healthcare professional supported adjustments of health expectations to fit couples’ everyday lives, it seemed to increase competence and autonomy to engage in diet and physical activity behaviors. Differing and mutual preferences for health behaviors existing within couples are also linked to their motivation for health behavior change. Lastly, couples’ perception of T2D as a health threat was identified as impacting their motivation to engage in health behaviors.

Consistent with SDT, relatedness was established through creating a non-judgmental environment and removing couples’ pressure to live up to the norms of perfect parenthood [35]. SDT posits that when patients and clinicians interact and medical advice is expected, it is important to establish relatedness before giving advice. Relatedness makes recipients more inclined to view the advice as informative rather than prescriptive [35]. The family wheel may have facilitated relatedness by enabling conversations about norms of health and parenthood through its diverse health-related topics. Consistent with studies on women with prior GDM, topics on mental health may facilitate motivation to engage in physical activity [50]. Also, role modeling healthy behaviors and home-based activities have been identified as potential effective intervention strategies for health promotion in families with small children [51]. Further, qualitative studies have highlighted expectations of prioritizing the family over oneself as a barrier to engaging in health behaviors [52,53]. Employing health visitors as the primary profession to deliver the intervention may have alleviated a non-judgmental environment due to their knowledge about health promotion in a family context [40]. Drawing from SDT, directing attention toward pursuits aligned with the family’s intrinsic interests, such as joy, might resonate with the family’s innate motivation, thereby fortifying their impetus to undertake health behaviors [35]. Thus, health visitors’ experience with realistic changes in a family setting may have spurred autonomous motivation by resonating with couples’ need to feel recognized while ensuring alignment with couples’ own interests.

Due to the need for manual registration and automated messages received in the LIVA app, couples’ mutual motivation for digital health coaching seemed to decrease due to its perceived incompatibility with family life. A recent review highlighted personalized goal setting and video coaching as highly acceptable features of digital health technology among women with prior GDM [28]. In the feasible MobileMums intervention, mothers reported that a personal connection to the intervention deliverer was facilitated by using first names, SMS texts, and possibly an initial face-to-face meeting [26]. Thus, our findings seem to be only partly consistent with the literature on digital interventions for postpartum mothers. Greenhalgh et al. argue that when an alternative solution to digital technology exists, the likelihood of withdrawal may increase [54]. Thus, the combination of home visits and digital coaching in the Face-it intervention may have decreased couples’ use due to feeling adequate support through home visits. Another reason for couples’ lack of interest in digital coaching was identified in a study on healthcare professionals’ perspectives [43]. In this study, it seemed that those health coaches who also conducted home visits generally preferred home visits due to the feeling that the online interaction compromised their relationships with couples [43]. Golob et al. found that women with lower educational attainment preferred face-to-face counseling vs. digital coaching [55], indicating that offering both digital coaching and home visits may be infeasible. Still, qualitative studies highlight the need for differentiated care to accommodate diverse needs among postpartum mothers and women with prior GDM [53,56]. Interestingly, more women than partners registered in the LIVA app, also indicating differing levels of interest in digital health coaching [32]. Partners may have felt underprioritized in the intervention due to their recruitment being based on their partners’ GDM diagnosis. Though partner support seems vital to increasing physical activity among mothers with small children [57], digital coaching may be more effective if offered to the woman and partner separately. For example, mothers in MobileMums indicated that they were satisfied with the sole focus on themselves as mothers [26]. Future studies should investigate how acceptability and adoption may be increased among women with prior GDM and their partners.

The current literature suggests that some women with prior GDM underestimate their risk of T2D development [58], which may decrease their motivation for health behavior change [59]. Similarly, for many couples in the current study, risk perception seemed to have a small or no effect on their motivation for health behavior change. Parson et al. explored risk among women with prior GDM and concluded that fear of T2D onset can work as a motivational factor for engaging in health behaviors [60]. Parson and colleagues proposed that diabetes risk may most optimally be addressed by considering both the woman’s personal beliefs and the socio-cultural context [60]. However, communicating about diabetes risk to couples was identified as troublesome by healthcare professionals in the development and delivery of the Face-it intervention [30,43]. Similar concerns have been documented among nurses, who have been found to under-communicate potential risks to avoid conflict with individuals they want to support [61]. Still, it seemed that some couples in our study had a shared interest in changing health behaviors in order to become a healthy family. Thus, ensuring a non-judgmental environment while providing information about the risk of diabetes (without it being normative or prescriptive) may be the most motivating way to create mutual, internalized motivation for health behaviors among couples. Still, more research is needed to understand how a motivational understanding of risk can be established in couples with a history of GDM.

We also identified how a woman’s perception of risk affected her partner’s interpretation of risk, with both positive and negative implications for their motivation for health behavior change. Although evidence of the partner’s role remains limited in the literature, in the context of couples with a history of GDM, partners seem to be motivated to support family health behaviors [25]. In our study, we found that partners did not consider themselves to be at risk, which, according to the interdependence theory, may have decreased their motivation for health behavior change [36]. A lack of motivation among partners may evoke an unsupportive home environment if partners persist in upholding unhealthy habits [62]. Although T2D risk among partners is addressed in the intervention manual for delivery of the Face-it intervention, it may be that healthcare professionals could have emphasized this shared risk of T2D even further to increase mutual motivation for health behavior change. Altogether, couples’ perceptions of diabetes risk seemed to have differing effects on their motivation for health behavior change, which should be investigated further.

### Strengths and Limitations

A strength of this study is our focus on couples, which allowed us to explore both individual and mutual motivations for health behavior change [42]. For example, we might not have identified the intrinsic motivation among couples for engaging in family-based activities if only the woman or the partner had been present. Also, our study sheds light on couples with a history of GDM as a highly relevant target group for T2D prevention, which is relevant to inform future health promotion interventions. The dual role that AT held as an intervention coordinator and the substantial engagement that AT had with the intervention could potentially have influenced the analysis and findings. Throughout the process, AT took reflective fieldnotes and documented preconceptions at the outset of the study. Also, AT worked closely with her co-authors, who read and provided feedback on three of the interview transcripts. HMA (third author) was not familiar with the Face-it intervention prior to overseeing the analytical process, which altogether increased the trustworthiness of the findings [63]. Our study also had limitations. For example, couples may have exaggerated their preference for family-based values due to the presence of their partner. Furthermore, couples sometimes found it difficult to recollect details about their experiences with the intervention, which may be a consequence of the interviews being performed up to 9 months after its completion. On the other hand, interviewing participants after they had completed the intervention rather than during the intervention allowed couples to reflect on the intervention as a whole. Directing questions at both women and their partners secured more equal involvement, facilitating more insights into partners’ views on the intervention.

We employed reflexive thematic analysis, which encourages researchers to continuously reflect upon and challenge their assumptions, to ensure transparent and trustworthy data collection and analysis [44]. For example, in interviews with couples, AT sought to uphold an explorative approach to health by asking: “What is health to you and your family?” SDT and interdependence theory helped to understand how motivation was established in couples by considering both individual and social dimensions of health behavior change. Combining these extensive theories increased sensitivity towards different interpersonal mechanisms. However, by focusing solely on motivation, we may have excluded other factors affecting couples’ health behavior change. For example, employing the Capability, Opportunity, Motivation-Behavior model by Michie et al. may have further advanced our findings [64].

## 5. Conclusions

Understanding the motivation for health behavior change among couples with a history of GDM gives a unique insight into how health promotion efforts may be tailored to the everyday lives of this target group. Across themes, individual tailoring to couples’ situational needs and beliefs seems vital to internalizing their motivation for health behavior change. Thus, to secure the engagement of the diverse group comprising women with prior GDM and their partners, we suggest targeting motivation through differentiated care. Knowledge gained from this study will contribute to the interpretation of the effects of the Face-it intervention and support the evidence base for health promotion among couples at increased T2D risk.

## Figures and Tables

**Table 1:** Couple characteristics (*n* = 14).

Characteristics	
	*n* = Couples
Interview setting Participants’ home Online with video	10 4
Child (ren) present during interview	
No Yes	8 6
Number of children within the couple 1 2	11 3
Time of interview after intervention ended (*n* = months)	
Mean (Range)	5.3 (1–9)
Age of the participating women (years)	
Mean (Range)	34.8 (25–42)
Age of the participating partners (years)	
Mean (Range)	35.8 (27–46)

## Data Availability

The qualitative data are unavailable due to privacy and ethical restrictions.

## Supplementary Materials

The following supporting information can be downloaded at: https://www.mdpi.com/article/10.3390/nu15183906/s1, Figure S1: The interactive dialogue tool, the family wheel; Table S1: Key questions in the interview guide for couple interviews; Table S2: Examples of the analytical process: Manifest content, interpretation, and theme.
