# Supplementary material for: Motivation for Behavior Change among Women with Recent Gestational Diabetes and Their Partners—A Qualitative Investigation among Participants in the Face-It Intervention
Source: Nutrients. 2023 Sep 7;15(18):3906. doi: 10.3390/nu15183906 (PMC10535498; doi:10.3390/nu15183906)

Table S1. Key questions in the interview guide for couple interviews

Transition to parenthood<br>• Can you tell me what it was like for you to become a family or having a second child?
Health concept<br>• Can you tell me what health is to you and your family and how this may have changed after having a baby?<br>• When do you feel healthy as a family?
Face-it intervention (prompt: family wheel and overview of intervention activities)<br>• What were your considerations on participating in the Face-it intervention?<br>• Can you describe what happened during the home visits?<br>• What were your thoughts about the home visits being delivered by a health visitor?<br>• What is your relationship to health technology, e.g., apps, activity trackers etc.?<br>• Can you tell me about your experiences with the LIVA app?<br>• Are there any parts of the interventions which you have seen as particularly useful or less useful?<br>• Can you tell me what you think you have gained from participating in the Face-it intervention?<br>• What were your thoughts on the intervention including both woman and partner?
Exercise<br>I would like you to think about aspects of the intervention which have been good and less good according to you throughout the intervention. To ensure that your views do not affect each other's replies, you should write down key words individually on post-its, after which we will discuss them together. You have five minutes.<br>Cue: Address the individual, who said the least.<br>What have you written on your notes and why?<br>Queue: Address the other individual<br>What do you think about the other's notes? Do you recognise it? Why? Why not?
Perspectives<br>• Can you tell me what you would have wanted to be different in the intervention?

Table S2. Examples of the analytical process: Manifest content, interpretation, and theme

Examples of manifest content	Interpretation	Theme
Most couples experienced a comfortable and supportive environment when healthcare professionals recognized the burdens of parenthood	When healthcare professionals took account of couples' personal challenges e.g. their psychosocial wellbeing, couples reported feelings of relatedness	The need to feel understood after delivery
Many couples were positive about the ability of healthcare professionals to explore and suggest realistic changes to their health habits	When healthcare professionals enabled couples to adapt their usual health habits to their needs, couples felt increased internalized motivation	Adjusting health expectations
Women and their partners described different uses of goal setting and interaction with the healthcare professional through the LIVA app	Individual preferences differed within couples, and digital health coaching accommodated these differences through individual goal setting and coaching	Individual and mutual preferences for health behaviors
Women expressed that healthcare professionals explained to them that it was not their fault that they developed GDM	Removing the shame of getting the GDM diagnosis allowed women to focus on future health behavior changes to reduce their T2D risk	The health threat of future T2D as a cue to action

Figure S1. The interactive dialogue tool, the family wheel

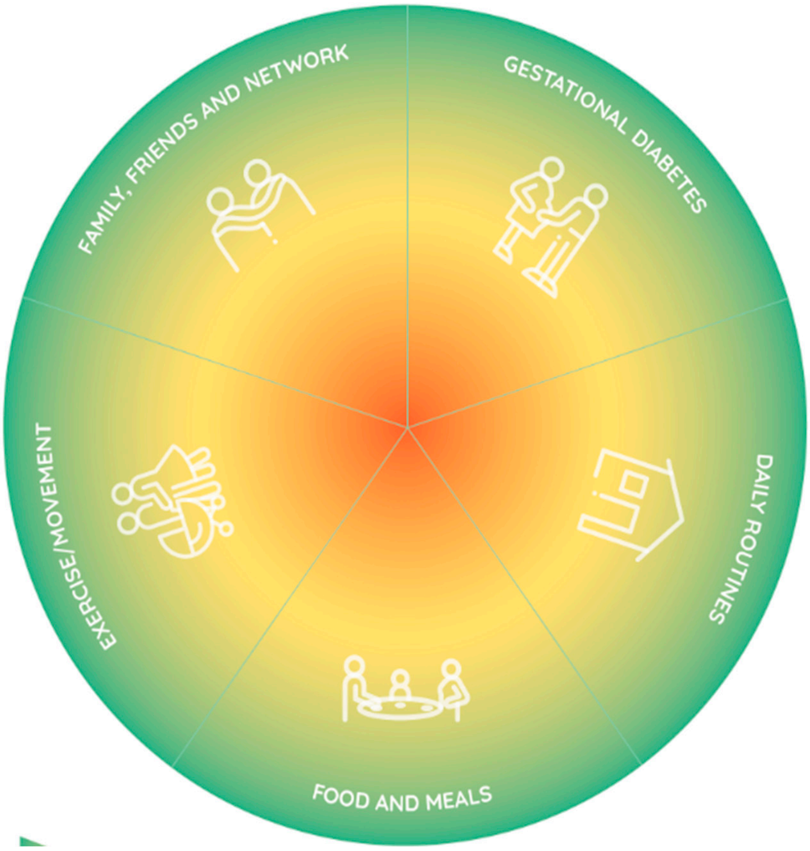

Supplement: Supplementary file 1 [file nutrients-15-03906-s001.zip › nutrients-2444020-supplementary.pdf]
